# Relationship between hyperemesis gravidarum and small-for-gestational-age in the Japanese population: the Japan Environment and Children’s Study (JECS)

**DOI:** 10.1186/s12884-016-1041-6

**Published:** 2016-08-26

**Authors:** Seiichi Morokuma, Mototsugu Shimokawa, Kiyoko Kato, Masafumi Sanefuji, Eiji Shibata, Mayumi Tsuji, Ayako Senju, Toshihiro Kawamoto, Koichi Kusuhara, Japan Environment & Children’s Study Group, Toshihiro Kawamoto, Hirohisa Saito, Reiko Kishi, Nobuo Yaegashi, Koichi Hashimoto, Chisato Mori, Fumiki Hirahara, Zentaro Yamagata, Hidekuni Inadera, Michihiro Kamijima, Ikuo Konishi, Hiroyasu Iso, Masayuki Shima, Toshihide Ogawa, Narufumi Suganuma, Koichi Kusuhara, Takahiko Katoh

**Affiliations:** 1 Research Center for Environmental and Developmental Medical Sciences, Kyushu University, Fukuoka, Japan; 2 Department of Obstetrics and Gynecology, Kyushu University Hospital, Kyushu University, Fukuoka, Japan; 3 Department of Cancer Information Research, Clinical Research Institute, National Kyushu Cancer Center, Fukuoka, Japan; 4 Department of Obstetrics and Gynecology, Graduate School of Medical Sciences, Kyushu University, 3-1-1 Maidashi, Higashi-ku, Fukuoka, 812-8582 Japan; 5 Department of Pediatrics, Graduate School of Medical Sciences, Kyushu University, Fukuoka, Japan; 6 Japan Environment and Children’s Study, UOEH Subunit Center, University of Occupational and Environmental Health, Kitakyushu, Fukuoka Japan; 7 Department of Obstetrics and Gynecology, School of Medicine, University of Occupational and Environmental Health, Kitakyushu, Fukuoka Japan; 8 Department of Environmental Health, School of Medicine, University of Occupational and Environmental Health, Kitakyushu, Fukuoka Japan; 9 Department of Pediatrics, School of Medicine, University of Occupational and Environmental Health, Kitakyushu, Japan

**Keywords:** Hyperemesis gravidarum, Small-for-gestational-age, Birth cohort

## Abstract

**Background:**

Small-for-gestational-age in infancy is a known risk factor not only for short-term prognosis but also for several long-term outcomes, such as neurological and metabolic disorders in adulthood. Previous research has shown that severe nausea and vomiting in early pregnancy (NVP) and hyperemesis gravidarum, which is an extreme form of NVP, represent risk factors for small-for-gestational-age birth. However, there is no clear consensus on this association. Thus, in the present study, we investigated the correlation between hyperemesis gravidarum and NVP on the one hand, and infant birth weight on the other, using data from the Japan Environment and Children’s Study (JECS).

**Methods:**

The data utilized in the present study were obtained from the JECS, an ongoing cohort study that began in January 2011. Our sample size was 8635 parent–child pairs. The presence or absence of severe NVP, hyperemesis gravidarum, and potential confounding factors were noted. A multivariable regression analysis was used to estimate risks for small-for-gestational-age birth, and the results were expressed as risk ratios and 95 % confidence intervals.

**Results:**

The risk ratios of small-for-gestational-age birth (95 % confidence interval) for mothers with severe NVP and those with hyperemesis gravidarum were 0.86 (0.62–1.19) and 0.81 (0.39–1.66), respectively, which represents a non-significant result.

**Conclusions:**

In our analysis of JECS data, neither severe NVP nor hyperemesis gravidarum was associated with increased risk for small-for-gestational-age birth.

## Background

There is a high incidence of nausea and vomiting in early pregnancy (NVP), reported at 35–91 % [1–4]. NVP can become severe in 0.3–3.6 % of cases, with hyperemesis gravidarum (HG) as an extreme form of NVP that is associated with weight loss [1–4]. The incidence of HG varies by country, and was reported at nearly 3.6 % in Japan [4].

The condition known as small-for-gestational-age (SGA) is a concern in infants, as it carries with it a multitude of risks, including a poorer life prognosis, neurological disorders, and metabolic diseases during adulthood [5, 6]. SGA is defined using the 10^th^ percentile for birth weight as the cutoff value [7, 8].

There are many risk factors for SGA, but most of these are not well understood. Extreme NVP may result in poor health during pregnancy, which can influence the prognosis of fetuses [9, 10], possibly leading to an increase in the risk of SGA birth [9, 11–13].

Recent systematic reviews suggest that HG increases the risk of low birth weight and SGA by 42 and 28 %, respectively [12]. Furthermore, severe maternal weight loss in early pregnancy, typically linked with extreme NVP, has been linked with growth restriction [9]. However, other reports have suggested that HG does not influence growth restriction [14, 15], birth weight [11, 16, 17], or risk for SGA [18]. Thus, there is as yet no clear consensus on this issue [11, 16, 17].

In the present study, we investigated the effect of severe NVP and HG (extreme NVP), with respect to the risk for SGA birth in the Japanese population.

## Methods

The data used in this study were obtained from The Japan Environment and Children’s Study (JECS), which is an ongoing cohort study that began in January 2011. The objective of the JECS is to determine the effect of environmental factors on children’s health.

More than 100,000 pregnant women were recruited over a period of approximately 3 years. The recruitment period ended in March 2014.

The pregnant women lived in one of the 15 study regions included in the JECS. The 15 regions were selected to cover wide geographical areas in Japan. We made contact with as many of these expecting mothers as possible. Either or both of the following two recruitment protocols were applied: 1) recruitment at the time of the first prenatal examination at cooperating health care providers, i.e., obstetric facilities (provider-mediated community-based recruitment), and/or 2) recruitment at local government offices issuing pregnancy journals, namely the Mother-Child Health Handbook, which is an official complimentary booklet that all expecting mothers in Japan are given when they become pregnant in order to receive municipal services for pregnancy, delivery, and childcare.

The JECS protocol was approved by the Review Board on epidemiological studies of the Ministry of the Environment, and by the Ethics Committees of all participating institutions. The JECS is conducted in accordance with the Helsinki Declaration and other nationally valid regulations, and with written informed consent from all participants. However, those who had difficulty filling out the questionnaire in Japanese or had other unavoidable circumstances preventing them from participating in the survey, such as being in their hometown at the time of childbirth, were excluded from the analysis [19, 20].

**Fig. 1:**
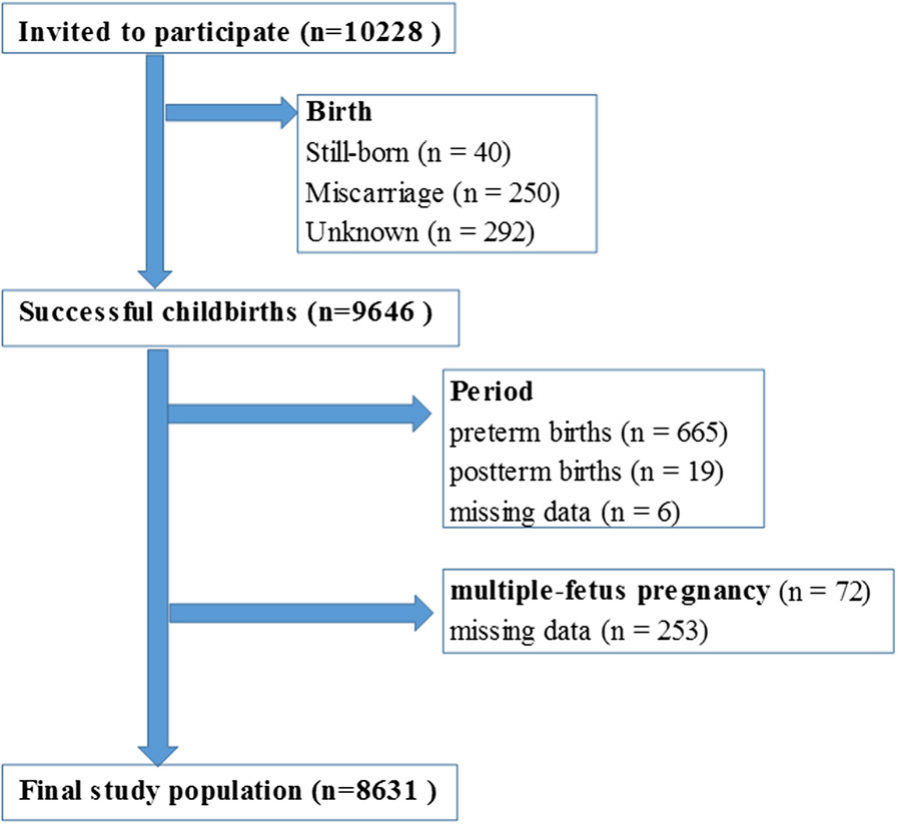
Participant inclusion flowchart

As of the end of 2011, a total of 9646 participants had successful childbirths. After excluding cases with missing data and preterm births, we analyzed the records of the remaining 8631 women who had single, full-term (37–42 weeks) pregnancies (Fig. 1). The present study is based on the data set “jecs-ag-ai-20131008”, which was released in October 2013.

Follow-up was conducted using a self-administered questionnaire. The questionnaires were completed during the first and second trimesters, as well as at 1 month postpartum. We obtained medical information from medical records transferred for examinations during the same time periods.

The questionnaires were designed to collect information on pregnancy and medical history as well as on confounding and modifying factors, such as social and lifestyle factors. We collected information on birth, such as the birth weight, from the transferred medical records.

The following question was included in the questionnaire for the second trimester to determine the status of HG: “Did you have morning sickness from conception until about week 12 of the pregnancy?” (1 = no, 2 = just nausea, 3 = vomiting, but was able to eat, 4 = vomiting, and was unable to eat). We thus defined the following groups for analysis: the “food intake group”, which included the women who answered 1, 2, or 3; the “no food” or severe NVP group, which included the women who answered 4; and the HG group, which was a subset of participants from the NVP group that included women with severe NVP and weight loss of >5 % from pre-pregnant weight in the first trimester.

The participants underwent ultrasound examinations during the first trimester, and these results were used to determine the expected date of delivery if there was more than a 7-day difference between this date and the date calculated from the last menstrual period. Birth weight was transferred from medical records, and SGA was concluded if the weight was below the 10^th^ percentile according to primiparous and multiparous birth size standards for both genders by gestational age in Japanese neonates [21].

The following covariates were included in the questionnaire for the first trimester: maternal age, pre-pregnancy body mass index (BMI), parity, smoking status, and alcohol consumption; the covariates of education and income were included in the questionnaire for the second trimester; the covariates of weight gain during pregnancy were calculated based on information from medical records.

### Statistical analysis

Based on the records of mothers of singletons delivered at full term, we evaluated the relationship between SGA and NVP, HG, factors related to the patient’s background, and social factors. Continuous variables were expressed as mean ± standard deviation (SD). We calculated crude relative risk ratios (RRs) and 95 % confidence intervals (CIs) using the chi-squared test. The interrelationship between patient background, social factors, and birth weight was evaluated by univariate analysis. Covariates of maternal age, pre-pregnancy BMI, weight gain during pregnancy, gestational age at birth, smoking, alcohol consumption, education, and income were included in the calculation of adjusted risk ratios. The adjusted relative RR was calculated using a log-binomial regression model. All statistical analyses were performed using SAS version 9.3 (SAS Institute Inc., Cary, NC, USA).

## Results

There were 880 patients (10.2 %) who experienced severe NVP, and 136 patients (1.6 %) who experienced HG. The mean age of participants, weeks of pregnancy at birth, and birth weight were 30.6 ± 5.02 years, 39.0 ± 1.14 weeks, and 3050.0 ± 371.32 g, respectively. The results of the univariate analysis are shown in Table 1. The adjusted risk ratios for mothers with a pre-pregnancy BMI of <18.5 kg/m^2^, mothers with a weight gain of <7 kg during pregnancy, and those who smoked were 1.58 (95 % CI, 1.32–1.90), 1.28 (95 % CI, 1.05–1.55), and 1.48 (95 % CI, 1.11–1.97), respectively, indicating a slightly higher risk of SGA birth. Moreover, the risk ratio was 0.60 (95 % CI, 0.43–0.85) for mothers with a pre-pregnancy BMI of >25 kg/m^2^, and 0.52 (95 % CI, 0.41–0.66) for mothers with a weight gain of >12 kg during pregnancy, indicating a lower risk of SGA birth.

**Table 1:** Characteristics of all parent-child pairs included in this study (*N* = 8631)

	No.	(%)	Missing data	No. of non-SGA births	No. of SGA births	% SGA	RR for SGA birth	95 % CI	
Mother’s age (years)									
19 or less	103	1.3	13	85	5	5.6	0.72	0.30	1.69
20–34^a^	5893	75.6	372	5093	428	7.8	(1.0)		
35 or more	1801	23.1	83	1610	108	6.3	0.81	0.66	0.99
missing	834		15	774	45				
Mother’s education									
> 12 years	5227	62.8	320	4558	349	7.1	0.97	0.82	1.14
≤ 12 years^a^	3098	37.2	135	2745	218	7.4	(1.0)		
missing	306		28	259	19				
Parity									
0^a^	3150	38.4	18	2893	239	7.6	(1.0)		
≥ 1	5043	61.6	27	4669	347	6.9	0.91	0.77	1.06
missing	438		438	-	-				
Pre-pregnancy body mass index									
< 18.5	1363	16.2	69	1153	141	10.9	1.58	1.32	1.90
18.5–24.9^a^	6212	73.7	346	5462	404	6.9	(1.0)		
≥ 25	856	10.2	34	788	34	4.1	0.60	0.43	0.85
missing	200		34	159	7				
Weight gain during pregnancy									
< 7 kg	1354	17.7	64	1162	128	9.9	1.28	1.05	1.55
7–12 kg^a^	4104	53.6	244	3560	300	7.8	(1.0)		
> 12 kg	2198	28.7	102	2011	85	4.1	0.52	0.41	0.66
missing	975		73	829	73				
Income									
< 4 million yen	3266	41.1	183	2854	229	7.4	1.07	0.90	1.27
4–8 million yen^a^	3836	48.3	208	3375	253	7.0	(1.0)		
> 8 million yen	843	10.6	53	732	58	7.3	1.05	0.80	1.39
missing	686		39	601	46				
Smoked during pregnancy									
No^a^	7991	94.5	455	7013	523	6.9	(1.0)		
Yes	462	5.5	15	401	46	10.3	1.48	1.11	1.97
missing	178		13	148	17				
Alcohol intake during pregnancy									
No^a^	7654	90.2	435	6710	509	7.1	(1.0)		
Yes	835	9.8	38	732	65	8.2	1.16	0.90	1.48
missing	142		10	120	12				

Data extracted from the Japan Environment and Children’s Study

*No.* number, *SGA* small-for-gestational-age, *RR* risk ratio, *CI* confidence interval

^a^Used as reference in the calculation of risk ratios

Tables 2 and 3 show the crude and adjusted risk ratios calculated using covariates such as the mother’s age, pre-pregnancy BMI, weight gain during pregnancy, parity, smoking and drinking, education, and income, to determine the effect of severe NVP or HG on the risk of SGA. The risk ratios for mothers with severe NVP and those with HG were 0.86 (95 % CI, 0.62–1.19) and 0.81 (95 % CI, 0.39–1.66), respectively, indicating a non-significant effect of NVP or HG on the risk for SGA birth.

**Table 2:** Risk for small-for-gestational-age (SGA) birth associated with severe nausea and vomiting in early pregnancy (NVP)

	Total number	No data on birth n (%)	Non-SGA birth n (%)	SGA birth n (%)	Crude		Confounder-adjusted	
					RR	95 % CI	RR	95 % CI
Severe NVP	880	48 (5.5)	773 (87.8)	59 (6.7)	0.98	0.75–1.27	0.86	0.62–1.19
No severe NVP^a^	7563	420 (5.6)	6625 (87.6)	518 (6.8)	(1.0)		(1.0)	
No data on NVP state	188	15 (8.0)	164 (87.2)	9 (4.8)				

The crude and adjusted risk ratios calculated using covariates such as the mother’s age, pre-pregnancy body mass index, weight gain during pregnancy, parity, smoking and alcohol consumption status, education, and income, to determine the effect of severe NVP on the risk of SGA birth

*RR* risk ratio, *CI* confidence interval

^a^Used as reference in the calculation of risk ratios

**Table 3:** Risk for small-for-gestational-age (SGA) birth associated with hyperemesis gravidarum (HG)

	Total number	No data on birth n (%)	Non-SGA birth n (%)	SGA birth n (%)	Crude		Confounder-adjusted	
					RR	95 % CI	RR	95 % CI
HG	136	8 (5.9)	119 (87.5)	9 (6.6)	0.97	0.51–1.83	0.81	0.39–1.66
No HG^a^	6393	331 (5.2)	5622 (87.9)	440 (6.9)	(1.0)		(1.0)	
No data on HG state	2102	144 (6.9)	1821 (86.6)	137 (6.5)				

The crude and adjusted risk ratios calculated using covariates such as the mother’s age, pre-pregnancy BMI, weight gain during pregnancy, parity, smoking and alcohol consumption status, education, and income, to determine the effect of HG on the risk of SGA birth

*RR* risk ratio, *CI* confidence interval

^a^Used as reference in the calculation of risk ratios

## Discussion

In our analysis of JECS data, neither NVP nor HG was associated with the risk for SGA birth. The incidence of HG was 1.6 %, which is lower than the 3.6 % incidence reported by the latest study in the general Japanese population [4], but within the range of 0.3–2.0 % reported by other studies [1–3]. In addition, the participants in our study reported an incidence of NVP of 10.2 %, which is lower than the 33 % incidence reported by Chortatos et al. [22]; the difference is likely related to the fact that we defined NVP based on self-reported accounts of reduced food intake.

Our study has a methodological limitation, because data regarding the severity of NVP were collected via a self-response questionnaire, while data regarding maternal weight loss were collected from the Mother-Child Health Handbooks and hospital records, and it is unknown whether participants required hospitalization for severe HG, how long severe NVP or HG persisted, and whether the condition reflected in the biochemical parameters.

Another limitation is the fact that the questionnaire was applied in the second trimester, but the questions themselves referred to early pregnancy; thus, there might be the risk of recall bias, resulting in an overestimation of the severity of NVP. However, we do not believe that this effect was significant, because the questionnaire was applied during the pregnancy period; moreover, the definition of HG was based on independent records of maternal weight loss.

A further limitation is related to the fact that our results were obtained based on the data regarding 136 cases of HG, which may be considered a small number in the context of an epidemiologic study. Nonetheless, given that the incidence of HG is expected to be under 2 %, and there is yet no consensus regarding the influence of HG on the risk for SGA birth, we believe that a sample size of 136 cases can ensure sufficient power to detect relevant trends, as some reports indicate that HG may increase the risk for SGA birth by up to 40 %; moreover, even if the power is low, the potential tendencies should be recognizable, because the confidence interval for our results is narrow.

Finally, another limitation of the study is related to the fact that the incidence of SGA birth in the group of mothers for whom weight gain information was missing was relatively high. Unfortunately, the reason for this higher incidence of SGA births cannot be assessed based on the data available to us. While it is possible that the characteristics of the mothers excluded from the study because of missing information on weight gain may have an influence on the results, we do not expect this influence to extend to the conclusions of our study.

Previous research demonstrating HG as a risk factor for SGA includes a study by Bailit et al., which showed that neonates born from mothers requiring hospitalization for HG were 125 g smaller compared to those born from mothers without such symptoms [11]. However, that study employed hospital admission rates for defining HG, which is a more subjective measure than is maternal weight loss. On the other hand, in other studies, which reported that HG leads to SGA birth [9, 10], the HG definition was based on maternal weight loss throughout pregnancy period; however, it was unclear whether the weight change was due to HG. In our study, the HG group included mothers with severe NVP (vomiting and not able to eat) and with weight loss of >5 % from pre-pregnant weight in the first trimester. Based on such a strict definition, our results showed that neither severe nor extreme NVP (i.e., HG) represented a risk factor for SGA birth.

The recent Norwegian Mother and Child Cohort Study reported that HG-exposed babies had slightly reduced birthweight, but there were no association between HG and SGA birth [18, 23], although it should be noted that no adjustment for weight gain was made, while adjusting for smoking status slightly increased the effect of HG. Further reports have suggested that HG does not influence birth weight [11, 16, 17]. Our results are in agreement with the findings of the studies that reported no relationship between HG and SGA birth; nevertheless, the relevance of adjusting for weight gain when evaluating the influence of HG should be noted, implying that the risk for SGA birth is reduced when sufficient weight gain is ensured during pregnancy.

It is important to note that both sets of studies (i.e., those concluding an effect and those concluding a lack of an effect) studied patients who required hospitalization. Even under these conditions, there is no conclusive evidence regarding the effect of HG on birth weight. Therefore, precise diagnostic criteria for HG should be developed for use in future investigations.

## Conclusions

Our results suggest that neither NVP nor HG affect birth weight. Despite the methodological limitations of the study, we believe that these results indicate that pregnant women need not be concerned about potential risk for SGA birth due to NVP or HG.

## Abbreviations

95 % CI: 95 % confidence interval
BMI: Body mass index
HG: Hyperemesis gravidarum
JECS: Japan Environment and Children’s Study
NVP: Nausea and vomiting in early pregnancy
RR: Risk ratio
SD: Standard deviation
SGA: Small-for-gestational-age
